# Fractional flow reserve vs angiography in non-ST- elevation myocardial infarction: long-term results of the FAMOUS-NSTEMI trial

**DOI:** 10.1093/eurheartj/ehaf925

**Published:** 2025-12-08

**Authors:** Colin Berry, Bethany Stanley, Patrycja Duklas, Jamie Layland, Keith G Oldroyd, Nick Curzen, Arvind Sood, Kanarath Balachandran, Raj Das, Shahid Junejo, Hany Eteiba, Mitchell Lindsay, Aadil Shaukat, Stuart Watkins, Ian Ford, Richard I S Good, Robert Henderson, Alex McConnachie

**Affiliations:** West of Scotland Heart and Lung Centre, Golden Jubilee National Hospital, Clydebank, UK; British Heart Foundation Glasgow Cardiovascular Research Centre, School of Cardiovascular and Metabolic Health, University of Glasgow, 126 University Place, Glasgow G12 8TA, UK; Robertson Centre for Biostatistics, University of Glasgow, Glasgow, UK; Robertson Centre for Biostatistics, University of Glasgow, Glasgow, UK; Peninsula Health, Peninsula Clinical School, Monash University, Melbourne, Australia; West of Scotland Heart and Lung Centre, Golden Jubilee National Hospital, Clydebank, UK; British Heart Foundation Glasgow Cardiovascular Research Centre, School of Cardiovascular and Metabolic Health, University of Glasgow, 126 University Place, Glasgow G12 8TA, UK; Faculty of Medicine, University of Southampton and University Hospital Southampton NHS Foundation Trust, Southampton, UK; Department of Cardiology, Hairmyres University Hospital, East Kilbride, UK; Department of Cardiology, Royal Blackburn Hospital, Blackburn, UK; Freeman Hospital, Newcastle Hospitals NHS Foundation Trust, Newcastle, UK; Department of Cardiology, South Tyneside and Sunderland NHS Foundation Trust, Sunderland, UK; West of Scotland Heart and Lung Centre, Golden Jubilee National Hospital, Clydebank, UK; West of Scotland Heart and Lung Centre, Golden Jubilee National Hospital, Clydebank, UK; West of Scotland Heart and Lung Centre, Golden Jubilee National Hospital, Clydebank, UK; West of Scotland Heart and Lung Centre, Golden Jubilee National Hospital, Clydebank, UK; Robertson Centre for Biostatistics, University of Glasgow, Glasgow, UK; West of Scotland Heart and Lung Centre, Golden Jubilee National Hospital, Clydebank, UK; British Heart Foundation Glasgow Cardiovascular Research Centre, School of Cardiovascular and Metabolic Health, University of Glasgow, 126 University Place, Glasgow G12 8TA, UK; Trent Cardiac Centre, Nottingham University Hospitals NHS Trust, Nottingham, UK; Robertson Centre for Biostatistics, University of Glasgow, Glasgow, UK

**Keywords:** Acute coronary syndrome, Non-ST-segment elevation myocardial infarction, Fractional flow reserve, Medical therapy, Coronary revascularization, Clinical outcomes

## Introduction

Complete revascularization may be considered for patients presenting with non-ST-segment elevation myocardial infarction (NSTEMI).^1,2^

Visual assessment of the angiogram may misclassify culprit lesion status,^3^ and a functional approach may guide revascularization.^4^ In stable coronary artery disease, fractional flow reserve (FFR) values >0.80 indicate that deferral of revascularization is safe^5^; however, in NSTEMI, microvascular dysfunction may limit hyperaemia,^6,7^ and plaque characteristics are prognostically important in non-flow limiting (FFR-negative) coronary disease.^8,9^

The FAMOUS-NSTEMI trial assessed FFR-guided vs angiography-guided management in 350 patients with NSTEMI.^10^ By 12 months, the percentage of participants treated by medical management only was higher in the FFR-guided group than in the angiography-guided group [40 (22.7%) vs 23(13.2%), difference 9.5% (95% CI 1.4%, 17.7%), *P* = .022; relative risk 1.72 (1.08, 2.82)].

We hypothesized that, compared with angiography-guided management, FFR-guided management in patients with recent NSTEMI would reduce coronary revascularization without increasing adverse clinical outcomes in the longer term.

## Methods

### Trial design

A prospective 1:1 randomized, controlled trial in 350 NSTEMI patients enrolled in six hospitals in the UK from October 2011 to May 2013 was undertaken,^10^ and a longer-term clinical outcomes analysis was prespecified.^10^

### Interventions

*FFR-guided group:* FFR ≤0.80 was an indication for revascularization by percutaneous coronary intervention (PCI) or coronary artery bypass surgery (CABG). FFR guidance was not recommended in angiographically severe or culprit lesions.

*Angiography-guided group and blinding:* in this group, FFR was measured but not disclosed.^10^

### Primary outcome

The prespecified primary outcome was spontaneous major adverse cardiovascular events (MACE) defined as cardiovascular death or hospitalization for myocardial infarction or heart failure. Cardiovascular death, stroke, and transient ischaemic attack were secondary outcomes.

### Sample size

We estimated that 5% of the population would experience a first MACE event annually from year two onwards (follow-up duration to 10 years), and at least 108 (31%) participants would experience a MACE. With 105 events, there would be 80% power to detect a hazard ratio (HR) of 0.58.

### Electronic health record linkage

Data for vital status and episodes of hospital care were obtained from NHS Scotland and NHS Digital in England. Standard clinical coding of medical records per the International Classification of Disease (ICD)-10 and OPCS Classification of Interventions and Procedures (OPCS)-4 were used.

### Statistical methods

Associations were assessed between randomized group and linkage health outcomes occurring after discharge from the index admission until the end of follow-up. Health outcomes were defined using the primary ICD-10 code recorded, and procedural events (CABG, PCI) were defined using all recorded OPCS-4 codes. Cardiovascular death was defined by the primary cause of death and using ICD-10 codes I00.x through to I99.x, where a suffix of ‘.x’ is used to indicate inclusion of all sub-codes within the given code range. MACE were defined as a composite of cardiovascular death or hospitalization for myocardial infarction (ICD-10 codes I21.x, I22.x) or heart failure (ICD-10 codes I50.0, I50.1, I50.9, I42.0, I42.9, I11.0, I25.5, I13.2, and I13.0). Coronary revascularization procedures included PCI, identified by OPCS-4 codes K49.x, K50.x, and K75.x, and CABG, identified by OPCS-4 codes K40.x–K46.x and K47.1. All randomized participants with linkage data available were included. The number of participants experiencing an event, person-years at risk, and event rate per 1000 person-years were calculated for the FFR-guided and standard care groups. The Cox regression model hazard ratio (HR), 95% CI, and corresponding *P*-value are presented for the FFR-guided group relative to the standard care group. All tests were two-tailed and assessed at the 5% significance level using R Studio version 4.0.0.

## Results

The duration of follow-up from the day after the index hospitalization discharge date until death or end of follow-up (data extract 31 March 2022), whichever occurred earliest, was 9.3 (9.0–9.8) years.

Of 350 randomized participants, 324 (93%) had complete follow-up, i.e. were successfully linked to their electronic health records. Of these participants (*n* = 324), 161 (49.7%) (mean age 62.5 years, 73.3% males) and 163 (50.3%) (mean age 62.1 years, 71.8% males) had been randomized to the FFR-guided and angiography-guided groups, respectively.

**Figure 1:**
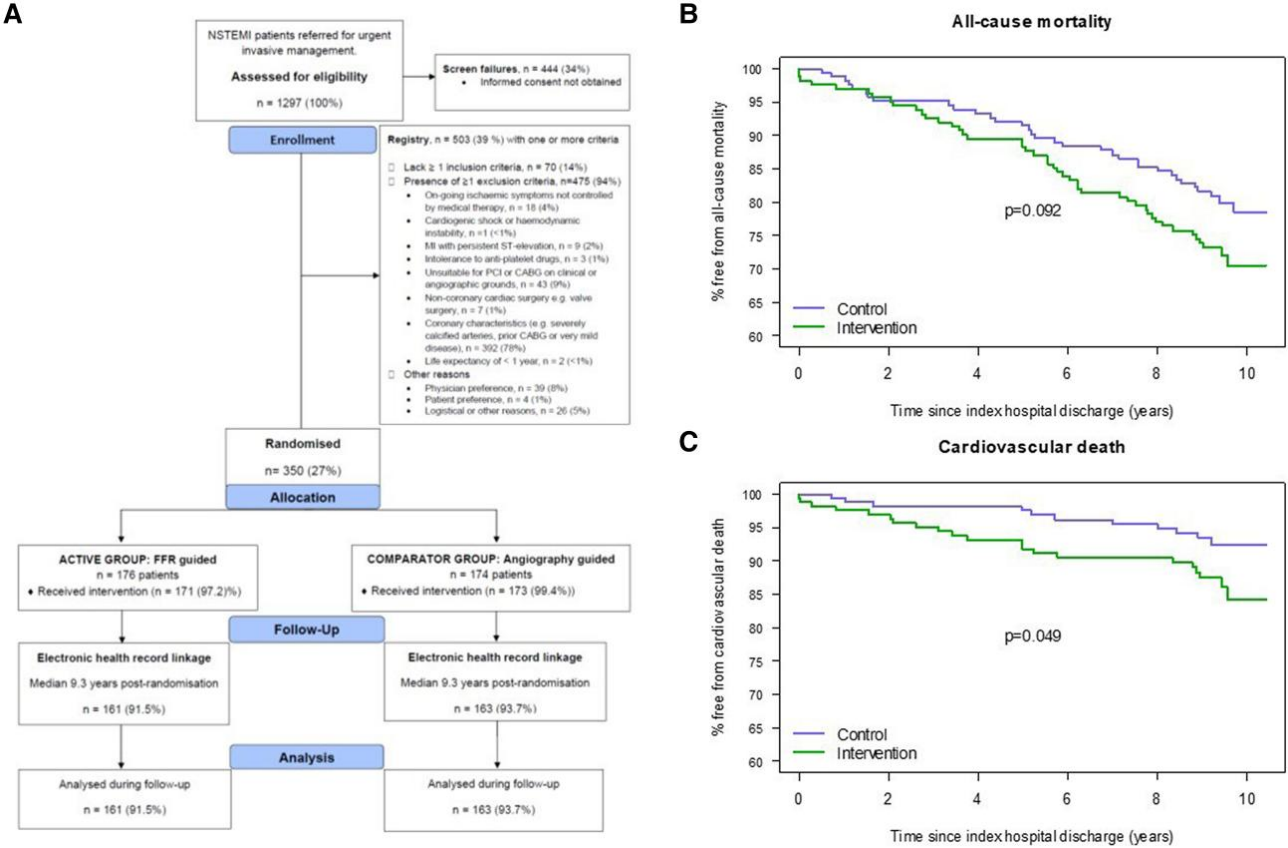
*A*) CONSORT flow diagram. Of the 350 randomized participants, 251 (72%) participants were enrolled in two hospitals in Scotland, and 99 (28%) participants were enrolled in four hospitals in England. *B*) Kaplan–Meier survival plot of time from index hospital discharge until death (all-cause) by randomized treatment group (*n* = 324 individuals; electronic health record linkage was achieved in 161 participants in the intervention group and 163 participants in the control group). Solid lines present the survival estimates. The *P*-value (*P* = .092) is from the log-rank test comparing the survival curve of each randomized treatment group. Population: all randomized patients with linkage data available during 9.3 (9.0–9.8) years follow-up. Intervention—FFR-guided group. Control—angiography-guided group. *C*) Kaplan–Meier survival plot of time from index hospital stay discharge until cardiovascular death by randomized treatment group during 9.3 (9.0–9.8) years follow-up. Solid lines present the survival estimates. The *P*-value (*P* = .049) is from the log-rank test comparing the survival curve of each randomized treatment group. Population: all randomized patients with linkage data available (*n* = 324; electronic health record linkage was achieved in 161 participants in the intervention group and 163 participants in the control group). Intervention (green)—FFR-guided group. Control (blue)—angiography-guided group

For the primary outcome (MACE), 45 of 161 (28.0%) participants in the FFR-guided group, and 38 of 163 (23.3%) participants in the angiography-guided group experienced a MACE [number with event/person-years at risk (rate per 1000 person-years): 45/1197 (37.6) vs 38/1284 (29.6); hazard ratio (95% confidence interval) 1.26 (0.82, 1.95); *P* = .288] (*Figure 1*).

The death rate was higher in the FFR-guided group [45/1337 (33.7) vs 33/1426 (23.1); 1.47 (0.94, 2.30); Cox regression *P* = .094; Kaplan–Meier log-rank test, *χ*^2^ (1, *N* = 324) = 2.83, *P* = .092]. The cardiovascular death rate was higher in the FFR-guided group [21/1337 (15.7) vs 11/1426 (7.7); 2.05 (0.99, 4.26); Cox regression *P* = .054; Kaplan–Meier log-rank test, *χ*^2^ (1, *N* = 324) = 3.88, *P* = .049].

Coronary revascularization by PCI or CABG after discharge was performed in 26/161 (16.1%) of the FFR-guided group vs 37/163 (22.7%) of the angiography-guided group [number with event/person-years at risk (rate per 1000 person-years): 26/1187 (21.9) vs 37/1208 (30.6); hazard ratio 0.72 (95% CI .43, 1.18); *P* = .193].

### Change in treatment plan post-randomization and clinical outcomes

The associations between change in the initial plan for revascularization (PCI or CABG) to medical management post-randomization and clinical outcomes were assessed.

MACE were not different between groups with or without a change in revascularization treatment decision post- vs pre-randomization, and coronary revascularization remained reduced (10/34 (29.4%) in the change group vs 35/127 (27.6%) in the no change group [number of individuals experiencing an event, person-years at risk, and event rate per 1000 years—10/253 (39.5) vs 35/944 (37.1); Cox hazard ratio (95% CI) 1.08 (0.54, 2.19); *P* = .824].

## Discussion

In NSTEMI patients followed for 10 years, compared with angiography-guided management, FFR-guided management did not reduce adverse cardiovascular outcomes.

The results support guideline recommendations against functional evaluation of an infarct-related coronary artery during the index procedure^1,2^ and highlight the uncertainty about culprit lesion classification in NSTEMI.^8^ The Complete-NSTEMI (NCT05786131) and COMPLETE-2 (NCT05701358) clinical trials will expand on these findings.

### Limitations

The trial was not powered for mortality endpoints and the between-group difference in cardiovascular deaths was not statistically significant.

## Declarations

### Data Availability

Data can be made available on reasonable request and with the agreement of the steering committee and sponsor.

### Pre-registered Clinical Trial Number

The clinical trial registration numbers are NCT01764334 and ISRCTN97489534, and the sponsor is the Golden Jubilee National Hospital, NHS Scotland.
